# Dataset of numerical modelling results of wave thrust on salt marsh boundaries with different seagrass coverages in a shallow back-barrier estuary

**DOI:** 10.1016/j.dib.2019.104197

**Published:** 2019-07-06

**Authors:** Carmine Donatelli, Neil K. Ganju, Tarandeep Singh Kalra, Sergio Fagherazzi, Nicoletta Leonardi

**Affiliations:** a Department of Geography and Planning, School of Environmental Sciences, Faculty of Science and Engineering, University of Liverpool, Roxby Building, Chatham St., Liverpool L69 7ZT, UK; b U.S. Geological Survey, Woods Hole Coastal and Marine Science Center, MA 02543, USA; c Integrated Statistics, Contracted to the U.S. Geological Survey, Woods Hole Coastal and Marine Science Center, MA 02543, USA; d Department of Earth Sciences, Boston University, 675 Commonwealth Avenue, Boston, MA 02215, USA

**Keywords:** Vegetation, COAWST, Wave thrust

## Abstract

This article contains data on the effects of seagrass decline on wave energy along the shoreline of Barnegat Bay (USA) previously evaluated in Donatelli et al., 2019. This study was carried out applying the Coupled-Ocean-Atmosphere-Wave-Sediment Transport (COAWST) numerical modelling framework to six historical maps of seagrass distribution. A new routine recently implemented in COAWST was used, which explicitly computes the wave thrust acting on salt marsh boundaries. The numerical modelling results are reported in terms of wind-wave heights for different seagrass coverages, wind speeds and directions. From a comparison with a numerical experiment without submerged aquatic vegetation, we show how the computed wave thrust on marsh boundaries can be reduced by seagrass beds.

**Table:** Specifications Table

Subject area	Geosciences
More specific subject area	Coastal hydrodynamics
Type of data	Table, figure
How data was acquired	Numerical simulations, COAWST modelling framework
Data format	Analysed data
Experimental factors	The seagrass coverages were exported from shape files provided by Lathrop et al. database (CRSSA) and added into the model.
Experimental features	The model was forced with tides and an idealized wind field for a neap-spring tidal cycle.
Data source location	Liverpool, United Kingdom
Data accessibility	http://doi.org/10.5281/zenodo.2647398
Related research article	Donatelli, C., Ganju, N.K., Kalra, T.S., Fagherazzi, S., and Leonardi, N., (2019). Changes in hydrodynamics and wave energy as a result of seagrass decline along the shoreline of a microtidal back-barrier estuary. Adv. In Water Resources. https://doi.org/10.1016/j.advwatres.2019.04.017

**Table:** 

**Value of the data** • The modelled wave thrust values can be used to evaluate how seagrass loss has influenced salt marsh lateral erosion in Barnegat Bay-Little Egg Harbor estuary over the last few decades. • This dataset can be used to make a comparisons with other coastal embayments to illustrate how the coastal protection functions of seagrass meadows change with the tidal range and water depth of the system. • Data could be used for investigation dealing with seasonal changes of seagrass coverage and associated changes in seagrass' coastal protection services.

## 1 Data

**Fig. 1:**
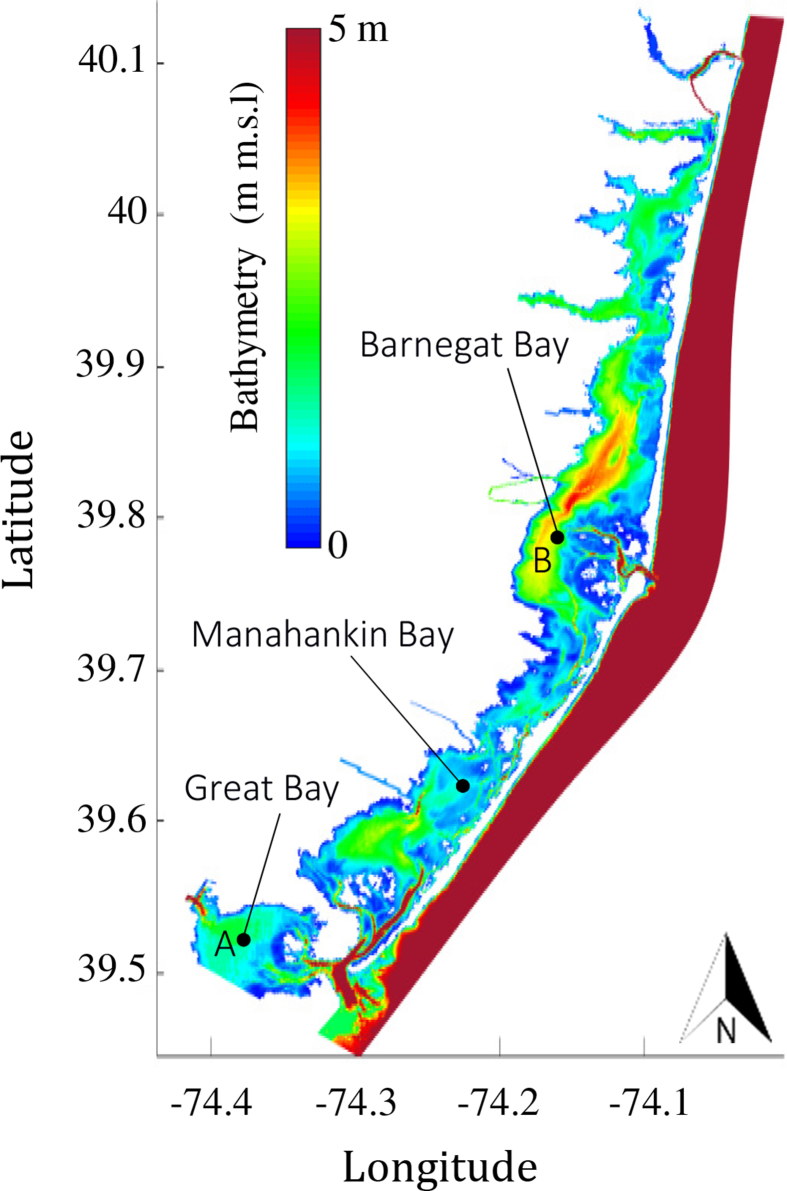
Bathymetry of Barnegat Bay-Little Egg Harbor estuary.

**Fig. 2:**
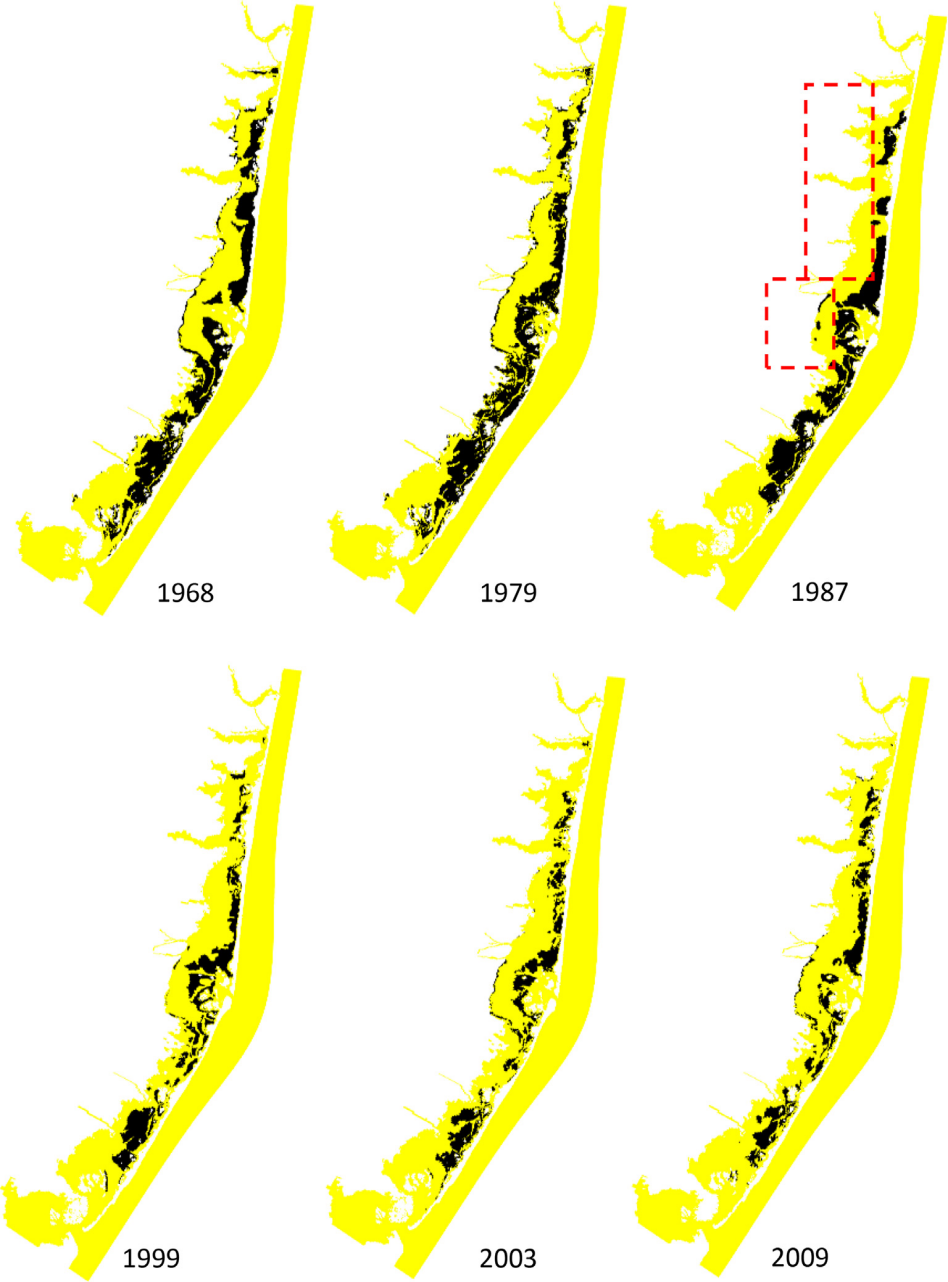
Seagrass coverages for different years. A different colormap is used to highlight SAV patches next to marsh boundaries.

**Fig. 3:**
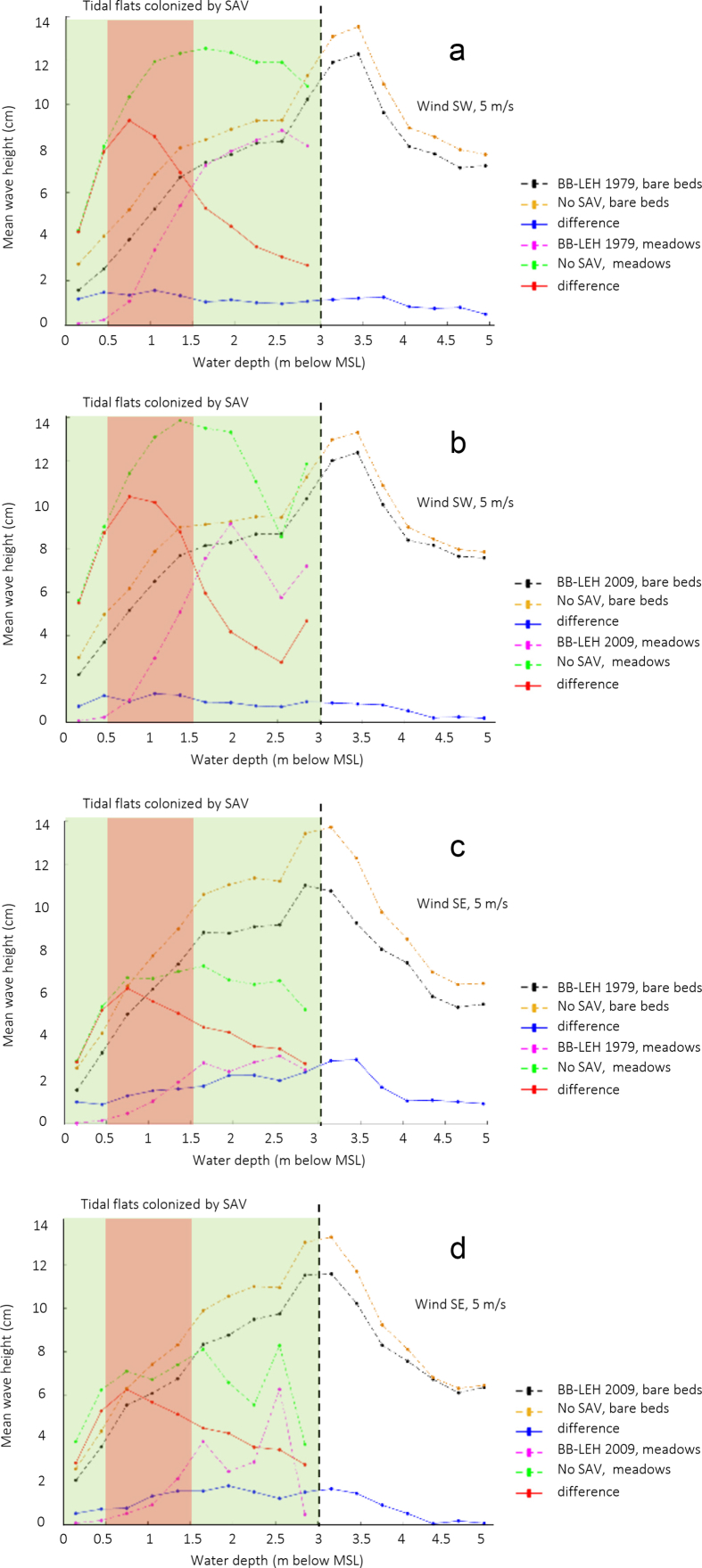
Mean wave height (cm) over bare beds and meadows as a function of water depth (m) for a wind blowing from south-west (a, b) and south-east (c, d) with a speed of 5 m/s. Panels a, c refer to seagrass distribution of 1979, while panels b, d refer to seagrass distribution of 2009; differences are made with respect to the no seagrass case. Water depth data are binned every 0.3 m.

**Fig. 4:**
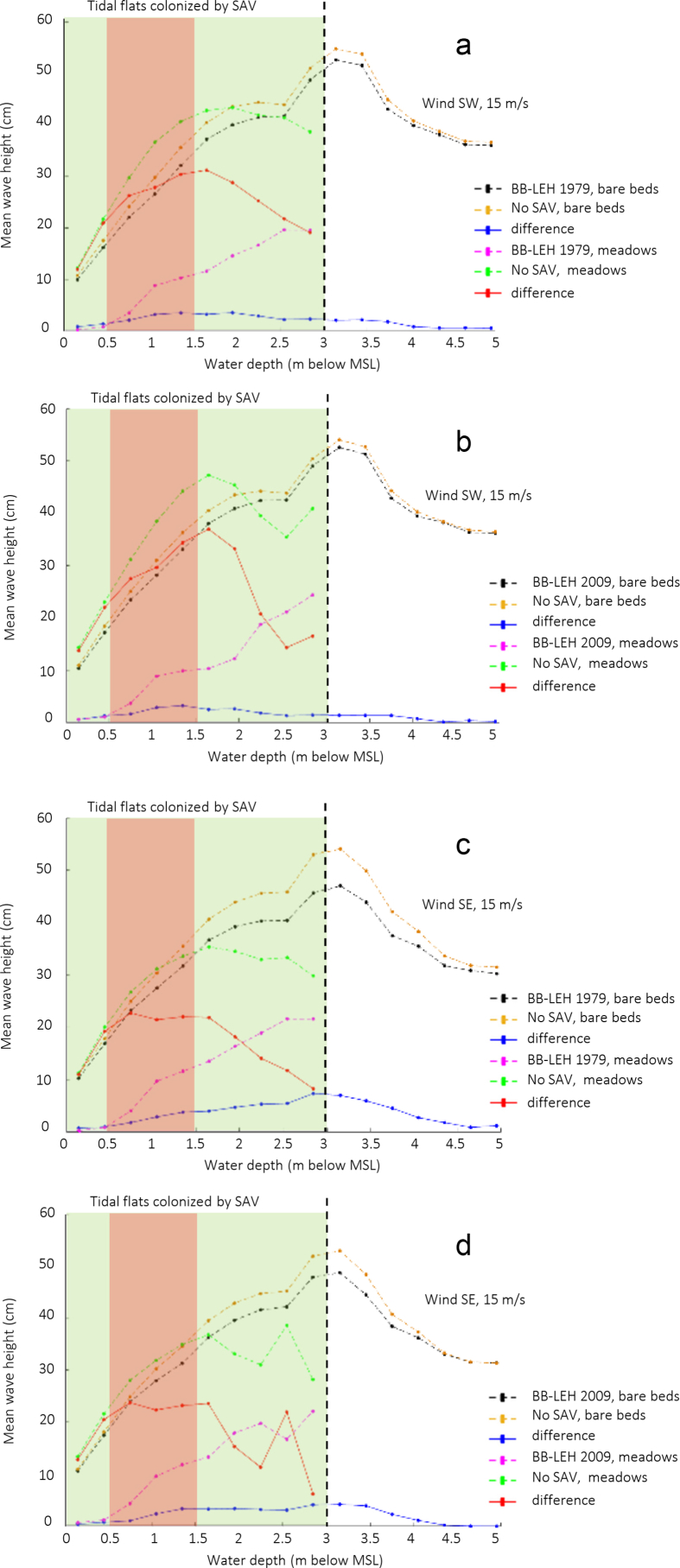
Mean wave height (cm) over bare beds and meadows as a function of water depth (m) for a wind blowing from south-west (a, b) and south-east (c, d) with a speed of 15 m/s. Panels a, c refer to seagrass distribution of 1979, while panels b, d refer to seagrass distribution of 2009; differences are made with respect to the no seagrass case. Water depth data are binned every 0.3 m.

**Fig. 5:**
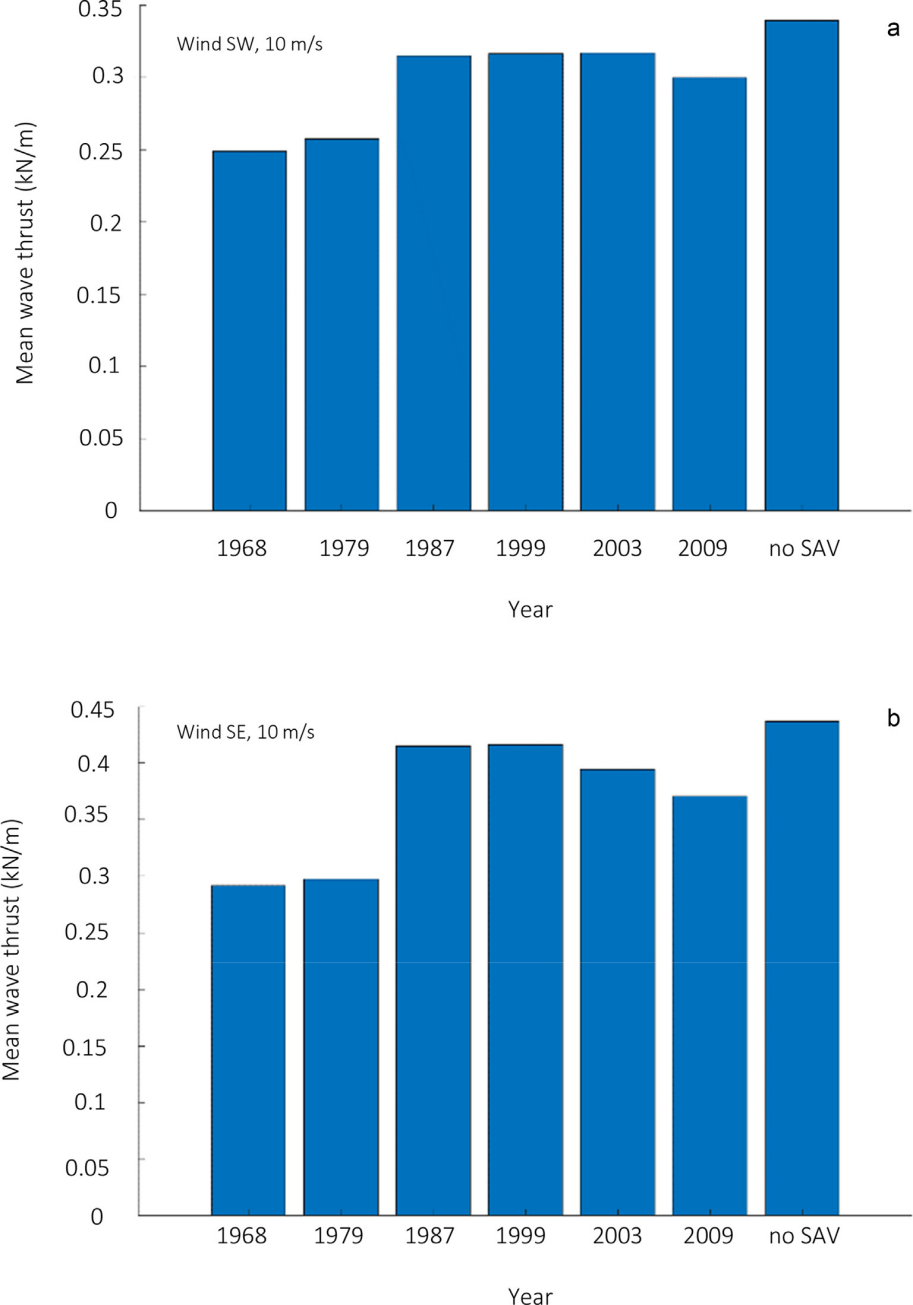
Mean wave thrust (kN/m) for each year for a wind blowing from south-west (a) and south-east (b) with a speed of 10 m $s^{-1}$in all the bay (Great Bay excluded).

**Fig. 6:**
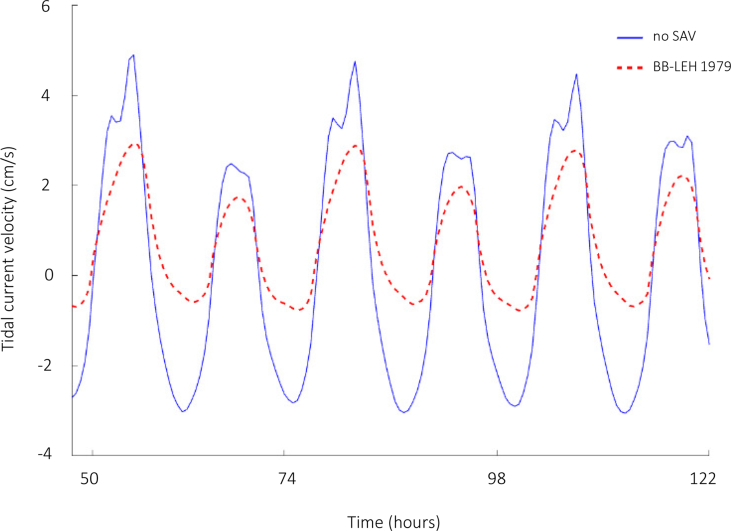
Tidal current velocity for six tidal cycles in a point of the bay (39.7923° N, 74.1715° W).

Numerical modelling results of wave thrust are presented here for the Barnegat Bay-Little Egg Harbor estuary (Fig. 1 and Fig. 2). We evaluated the influence of seagrass beds on locally generated waves for winds of 5 and 15 m/s blowing from south-east and south-west. Fig. 3, Fig. 4 present the distributions of mean wave height as a function of water depth in the non-seagrass case and for the scenarios with maximum (year 1979) and minimum (year 2009) seagrass coverage. In addition, the effect of seagrass decline on the mean wave thrust is presented in a bar chart (Fig. 5) for a wind of constant speed (10 m/s). The mean wave thrust is defined as the mean value computed throughout the entire simulation period along the marsh boundaries. The main result is that seagrass presence can attenuate the wave thrust by 28% for a wind blowing from the south-west direction and by 33% for a wind blowing from the south-east direction (Fig. 5). The influence of seagrass meadows on tidal asymmetry measured at 39.7923° N, 74.1715° W is depicted in Fig. 6. The flood and ebb peak velocities are increased respectively by 40% and 64% with seagrass removal in that point.

## 2 Experimental design, materials, and methods

The hydrodynamics of the system was simulated using the COAWST (Coupled-Ocean-Atmosphere-Wave-Sediment Transport Modeling System) modeling framework [2]. Details of the numerical model set-up are available in Donatelli et al. [1], and Defne & Ganju [3]. The wave thrust (the integral along the vertical of the dynamic pressure of waves) acting on marsh boundaries is explicitly computed by the model following Tonelli et al. [4], and Leonardi et al., [5]. The COAWST modelling framework is built to allow the user to select any combination of the main three models (ROMS, SWAN and WRF). The user needs to list C-preprocessing options in a header file to select the models, to couple them and to activate any specific individual option available for each model. Specifically, the new wave thrust routine (Supporting Information of Leonardi et al. [5], now implemented into COAWST) is activated by the following flags:

# define MARSH_WAVE_EROSION.
# define MARSH_WAVE_THRUST.
# undef MARSH_SED_EROSION.
and activating the new vegetation module recently implemented in COAWST by Beudin et al. [6]:
# define VEGETATION.
# ifdef VEGETATION.
# undef ANA_VEGETATION.
# define VEG_DRAG.
# ifdef VEG_DRAG.
# define VEG_FLEX.
# define VEG_TURB.
# endif.
# define VEG_SWAN_COUPLING.
# ifdef VEG_SWAN_COUPLING.
# define VEG_STREAMING.
# endif.

In numerical models, the simplest method to simulate the influence of plants on the mean flow is to increase the bottom roughness coefficient [7], [8]. However, this method cannot properly represents the three-dimensional influence of vegetation on the mean and turbulent flow [9], [10]. The new flow-vegetation module affects the flow field through plant posture-dependent three-dimensional drag, in-canopy wave-induced streaming, and production of turbulent kinetic energy and enstrophy for the vertical mixing parameterization. The vegetation drag force is computed using a quadratic drag law and the effect of plant flexibility in reducing drag is computed defining an effective blade length following the approach of Luhar & Nepf [11]. The selected turbulence model is the k–ε scheme which accounts for extra dissipation and turbulence kinetic energy production due to vegetation [12]. The wave dissipation due to vegetation is calculated by the model modifying the source term of the action balance equation following the formulation of Mendez & Losada [13], and implemented in SWAN by Suzuki et al., [14].

The presence of marsh is felt by the wave thrust routine through the variable marsh_mask, which is specified in the initial condition file. The variable marsh_mask is defined by a matrix with 0 and 1, where marsh pixels have a value of 1. Finally, the user needs to create a vegetation input file where mass density, number of vegetation types and mechanical properties of plants are listed:

NVEG == 1 ! Number of submerged aquatic vegetation types.
CD_VEG == 1.0d0 ! Drag coefficient for each vegetation type.
E_VEG == 1.0d9 ! Young's Modulus for each vegetation type.
VEG_MASSDENS == 700.0d0 ! Mass density for each vegetation type.
VEGHMIXCOEF == 0.1d0 ! Additional horizontal viscosity coefficient at the edge of a vegetation patch.
KFAC_MARSH == 0.6d-5 ! Marsh erosion factor depends on sediment cohesive properties.
SCARP_HGHT == 0.2d0.
! Logical switches (TRUE/FALSE) to activate writing of vegetation fields.
! into HISTORY output file: [1:NVEG,Ngrids]
Hout(ipdens) == F ! Plant_density Density of the plant for each vegetation.
Hout(iphght) == F ! Plant_height Height of the plant for each vegetation.
Hout(ipdiam) == F ! Plant_diameter Diameter of the plant for each vegetation.
Hout(ipthck) == F ! Plant_thickness Thickness of the plant for each vegetation.
Hout(ipagbm) == F ! Plant_agb Above ground plant biomass.
Hout(ipbgbm) == F ! Plant_bgb Below ground plant biomass.
Hout(idWdvg) == F ! Dissip_veg Wave dissipation due to vegetation.
Hout(idTims) == T ! marsh_mask masking for getting thrust due to waves.
Hout(idTtot) == T ! Thrust_total Total thrust due to waves.
Hout(idTmfo) == F ! marsh_flux_out Marsh flux out.
Hout(idTmmr) == F ! marsh_retreat Amount of marsh retreat from all four directions.
Hout(idTmsc) == F ! marsh_scrp_height Amount of marsh retreat from all four directions.

Different scenarios were considered for the wind forcing characterized by winds of constant speed (5, 10 and 15 m/s) blowing from south-west and south-east for the entire period of simulation. Seagrass aerial extent and vegetation parameters are listed in Table 1 and Table 2.

**Table 1:** Ratio between seagrass extent and basin area for each year.

Year	Vegetated bed/Basin area
1968	0.3
1979	0.31
1987	0.27
1999	0.18
2003	0.16
2009	0.16

**Table 2:** Vegetation parameters.

	Canopy height (cm)	Stem density (shoots/m2)	Mass density (kg/m3)	Young's module (kN/mm2)
Salt marsh	50	248	700	1
Seagrass	20	251, 600, 900	700	1
